# Identification of factors associated with residual malaria transmission using school-based serological surveys in settings pursuing elimination

**DOI:** 10.1186/s12936-022-04260-0

**Published:** 2022-08-21

**Authors:** Jean Marius Rakotondramanga, Inès Vigan-Womas, Laura C. Steinhardt, Aina Harimanana, Elisabeth Ravaoarisoa, Tsikiniaina L. Rasoloharimanana, Seheno Razanatsiorimalala, Amy Wesolowski, Milijaona Randrianarivelojosia, Benjamin Roche, Andres Garchitorena

**Affiliations:** 1 Epidemiology and Clinical Research Unit, Institut Pasteur de Madagascar, Antananarivo, Madagascar; 2 IRD, Sorbonne Université, UMMISCO, 93143 Bondy, France; 3 Sorbonne Université, ED 393, Paris, France; 4 MIVEGEC, Université Montpellier, CNRS, IRD, Montpellier, France; 5 Immunology of Infectious Diseases Unit, Institut Pasteur de Madagascar, Antananarivo, Madagascar; 6 Immunophysiopathology and Infectious Diseases Department, Institut Pasteur de Dakar, Dakar, Senegal; 7 Malaria Branch, Division of Parasitic Diseases and Malaria, Center for Global Health, Centers for Disease Control and Prevention, Atlanta, GA USA; 8 Faculty of Sciences, University of Antananarivo, Antananarivo, Madagascar; 9 Unité de Parasitologie, Institut Pasteur de Madagascar, Antananarivo, Madagascar; 10 Department of Epidemiology, Johns Hopkins Bloomberg School of Public Health, Baltimore, MD USA; 11 Faculté Des Sciences, Université de Toliara, 601 Toliara, Madagascar

**Keywords:** Malaria, *Plasmodium falciparum*, Antibody, Seroprevalence, AMA1, Spatial analysis, Cluster, Epidemiology, Madagascar

## Abstract

**Background:**

Targeted research on residual malaria transmission is important to improve strategies in settings pursuing elimination, where transmission reductions prove challenging. This study aimed to detect and characterize spatial heterogeneity and factors associated with *Plasmodium falciparum* infections and exposure, *P. falciparum* apical membrane antigen 1 (PfAMA1) antibody (Ab) response, in the Central Highlands of Madagascar (CHL).

**Methods:**

From May to July 2014, a cross-sectional school-based survey was carried out in 182 *fokontany* (villages) within 7 health districts of the CHL. Rapid diagnostic tests (RDTs) and a bead-based immunoassay including PfAMA1 antigen biomarker were used to estimate malaria prevalence and seroprevalence, respectively. Local Moran’s I index was used to detect spatial “hotspots”. Remotely sensed environmental data—temperature, vegetation indices, land covers, and elevation—were used in multivariable mixed-effects logistic regression models to characterize factors associated with malaria infection and cumulative exposure.

**Results:**

Among 6,293 school-children ages 2–14 years surveyed, RDT prevalence was low at 0.8% (95% CI 0.6–1.1%), while PfAMA1 Ab seroprevalence was 7.0% (95% CI 6.4–7.7%). Hotspots of PfAMA1 Ab seroprevalence were observed in two districts (Ankazobe and Mandoto). Seroprevalence increased for children living > 5 km from a health centre (adjusted odds ratio (OR) = 1.6, 95% CI 1.2–2.2), and for those experiencing a fever episode in the previous 2 weeks (OR 1.7, 95% CI 1.2–2.4), but decreased at higher elevation (for each 100-m increase, OR = 0.7, 95% CI 0.6–0.8). A clear age pattern was observed whereby children 9–10 years old had an OR of 1.8 (95% CI 1.2–2.4), children 11–12 years an OR of 3.7 (95% CI 2.8–5.0), and children 13–14 years an OR of 5.7 (95% CI 4.0–8.0) for seropositivity, compared with younger children (2–8 years).

**Conclusion:**

The use of serology in this study provided a better understanding of malaria hotspots and associated factors, revealing a pattern of higher transmission linked to geographical barriers in health care access. The integration of antibody-assays into existing surveillance activities could improve exposure assessment, and may help to monitor the effectiveness of malaria control efforts and adapt elimination interventions.

**Supplementary Information:**

The online version contains supplementary material available at 10.1186/s12936-022-04260-0.

## Background

Malaria remains one of the most important causes of morbidity and mortality in sub-Saharan Africa. While many countries in the African region have the potential to eliminate malaria in the medium or long term—malaria case incidence reduced from 363 to 225 cases per 1000 populations at risk in 2000 and 2020, respectively—Madagascar has still been aiming to improve case management for at least 95% of diagnosed cases and to ensure the permanent availability of diagnostic-and-treatment tools for 95% of health facilities since 2013 [1, 2]. Madagascar has a highly heterogeneous distribution of malaria transmission, with areas of high transmission in the east and west coast of the island, and areas of very low and low transmission in the Central Highlands (CHL) and surrounding Fringes areas, respectively [3, 4]. The National Malaria Control Programme (NMCP) has targeted five districts in the highlands for malaria elimination, with the goal to reach zero deaths, and to extend the number of districts targeted for elimination from 5 in 2018 to 13 by 2022, mainly in CHL and surrounding Fringes areas. However, progress so far has been elusive [5].

Characterizing malaria transmission intensity in near-elimination settings using passive surveillance and standard diagnostic methods can be challenging, as asymptomatic infections can outnumber symptomatic infections and are hard to detect with malaria rapid diagnostic tests (RDTs) only [6–8]. Yet, asymptomatic cases with low detectable levels of parasitaemia can constitute potential reservoirs for malaria [6, 8, 9]. In these settings, serological assays for antibody detection can be a powerful tool for estimating cumulative exposure in addition to RDTs and microscopy during large-scale surveillance, such as Malaria Indicator Surveys [10, 11]. Field studies have also shown that the predominant immunoglobulin G (IgG) subclass profiles of *Plasmodium falciparum* are influenced by age and exposure to infection [12]; in particular, IgG-specific antibody responses to *P. falciparum* merozoite antigens—the apical membrane antigen 1 (PfAMA1) and the 19 kDa C-terminal region of the merozoite surface protein 1 (PfMSP1-19)—and other blood-stage antigens can be good biomarkers of *P. falciparum* exposure in populations with low immunity such as children less than 15 years of age [13–15]. Thus, antibody responses against *P. falciparum* antigens, such as PfAMA1, can be particularly useful and informative to differentiate individuals based on their cumulative exposure, and to aid in characterizing factors associated with spatial heterogeneity in near-elimination settings [16, 17].

In moderate or low malaria transmission settings, characterizing malaria prevalence can yield somewhat homogeneous patterns at higher levels of spatial analysis. Yet, fine-scale population-based parasitaemia data can reveal local spatial heterogeneity in areas previously assumed to have uniform transmission [18]. Country-level surveys like the Demographic and Health Surveys (the finest resolution dataset used for Madagascar) have an average resolution of one cluster per 1000 km^2^ approximately [19]. Data at more granular levels can help to elucidate factors influencing malaria transmission like climate (including temperature, rainfall) and environmental factors (vegetation, elevation, and land covers), guiding district-scale programmatic efforts to control malaria [18]. Such data, when analysed with appropriate methods, can allow identification of malaria transmission hotspots and their characteristics [20]**,** therefore, allowing targeting of transmission residual pockets; which is critical in settings pursuing elimination.

After several years of blanket spraying in the CHL, more targeted indoor residual spraying (IRS) has been applied selectively to epidemic-prone areas since 2003 and insecticide-treated mosquito nets (ITNs) are regularly distributed to the population in mass campaigns into these settings [21]. The World Health Organization (WHO) has recommended at least one ITN per household; in the CHL, ITN ownership and access—the proportion of household members with access to an ITN—were 25% and 16% in 2013, respectively, both the lowest in the country [22]. Moreover, in order to detect and treat cases early, NMCP has implemented the systematic use of RDTs since 2010 for all suspected malaria cases with fever (axillary temperature > 37.5 °C) or history of fever in 2 weeks, but such strategy misses low-density asymptomatic infections that can still contribute to transmission in near elimination settings [6, 23–25].

In 2014, a cross-sectional school-based survey was carried out in seven districts of the CHL and surrounding Fringes areas to better characterize malaria transmission via use of serological markers of *P. falciparum* exposure [11]. Results showed that the ability of annual parasite incidence estimates using health facility routine data of malaria-confirmed patients by RDT to characterize malaria transmission declined at lower transmission levels as compared with a serological approach [11]. Using this dataset, this study expands on the previous analyses and aims (1) to characterize the spatial heterogeneity of malaria transmission intensity and to detect hotspots of both *P. falciparum* infection prevalence and PfAMA1 antibody (Ab) seroprevalence in CHL and surrounding Fringes; and (2) to identify sociodemographic, climatic, and environmental associated factors with such heterogeneity in malaria infection and exposure. These were not elucidated in Steinhardt et al. [11].

## Methods

### Study area

**Fig. 1:**
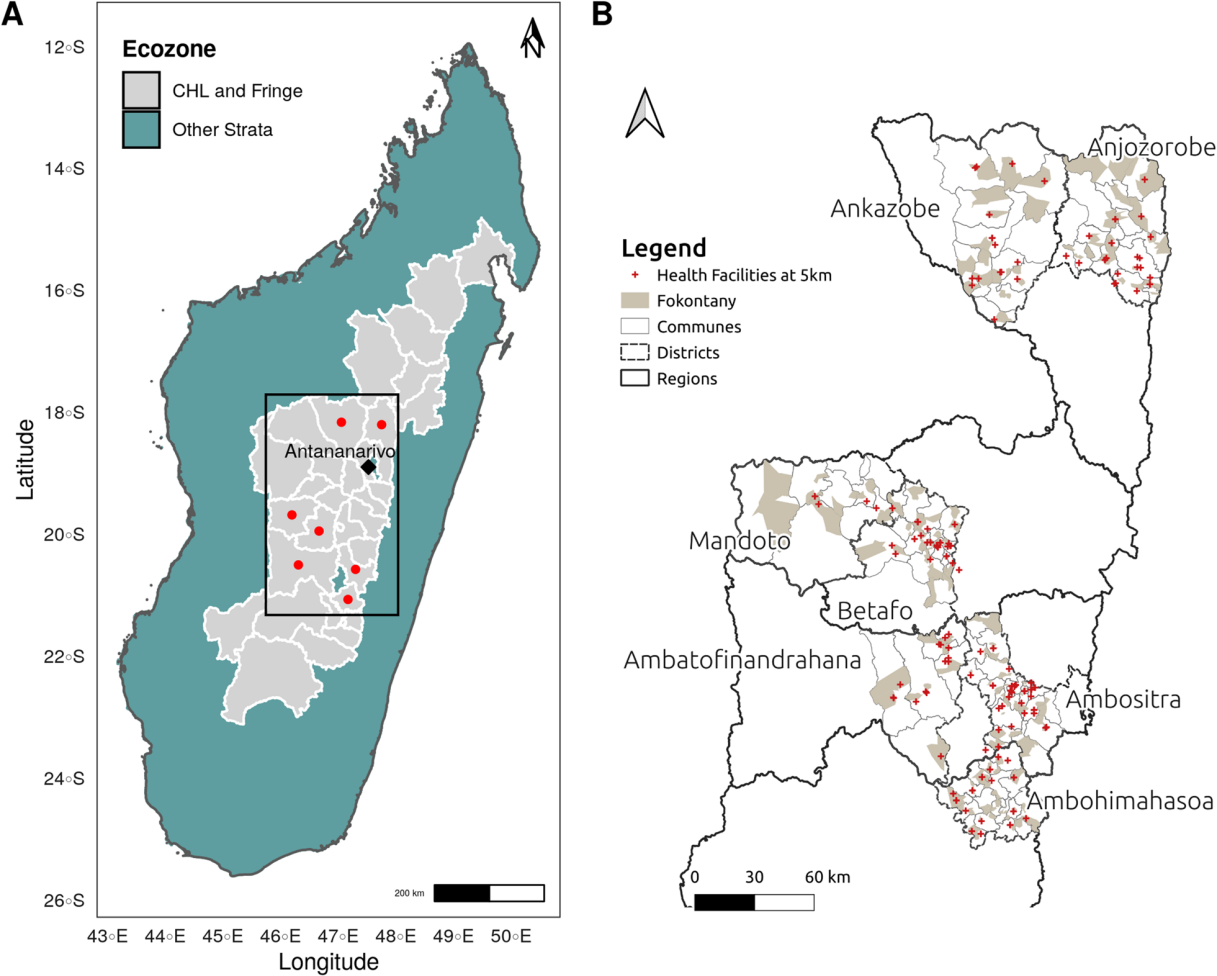
Map of the study area in the Central Highlands (CHL) of Madagascar with the locations of investigated *fokontany*. **A** Lower strata of malaria infection risk, defined by Howes et al. [3], in the CHL and Fringes areas are shown in the grey shaded area and higher-risk strata are shown in blue-green. The seven investigated districts are indicated in red and the black diamond represents the capital of Madagascar, Antananarivo. **B** *Fokontany* boundaries of investigated schools are shaded in grey; communes (a group of *fokontany*) in grey lines; districts in dashed-black lines; and the nearest health facilities (at < 5 km) from the *fokontany* centroid coordinates are marked by red crosses [27]

This study was carried out in 2014 in seven health districts from the CHL and surrounding Fringes areas (Fig. 1A). The health districts targeted were Ankazobe and Anjozorobe to the north of the capital city Antananarivo; Ambatofinandrahana, Ambohimahasoa, Ambositra, Betafo, and Mandoto to the south. The CHL and surrounding Fringes areas are characterized by unstable and episodic malaria transmission. Data from routine surveillance suggested that these areas had the lowest incidence in the country during 2013–2014, as measured by RDT [26], with more than 90% of the malaria infections occurring in the area due to *P. falciparum* [3]. In 2013, malaria prevalence in children aged 6–59 months, characterized via microscopy, was 0.7% and 2.5%, respectively, in CHL and surrounding Fringes areas [22]. While transmission is low, previous analyses of routine malaria surveillance data have indicated that substantial heterogeneity in malaria transmission exists in the CHL [4], which needs fine-scale risk and exposure assessment to adapt and improve malaria elimination efforts [3].

### Data collection

#### Survey and biological sample collection

To better understand and detect spatial heterogeneity in *P. falciparum* infection in the CHL and surrounding Fringes areas, a school-based seroepidemiological study was carried out from May to July 2014. The study has been described in detail by Steinhardt et al. [11]. Briefly, a cross-sectional survey using bead-based serological multiplex analyses was implemented in low-transmission and elimination settings [17]. In the seven sampled districts, all geographically accessible communes that presented no known safety issues for the study were surveyed, for a total of 93 communes out of 107 (Fig. 1B): for each selected commune, one primary school in proximity of a health facility (within 5 km distance) and one farther away (> 10 km) were selected. Overall, 182 *fokontany* (the smallest administrative unit,  ~ villages) were considered for investigation (Fig. 1B). Thirty children under 15 years old were selected randomly per school. This age group has been found to reflect populations with low immunity [14, 28, 29]. For each selected child, a questionnaire on demographics, residence, recent symptoms and trips (outside the commune), and household (or community) control measures (bed net use, IRS) was administered to their parent (or guardian).

Malaria RDTs [CareStart Malaria RDT, HRP2/pLDH (Pf/PAN) Combo; Access Bio] were performed with finger prick blood to detect malaria infections in all selected children. Children with a positive RDT were treated with artesunate-amodiaquine with age-appropriate doses, as recommended by national guidelines. Additionally, capillary blood was collected for all children in microvette tubes (Microvette 500 Z-Gel; Sarstedt) for later serological analyses. Collected samples were transported to Institut Pasteur de Madagascar’s immunology laboratory in Antananarivo and stored at − 20 °C until used [11].

#### Serological data

Laboratory serological analyses were conducted using 5 *P. falciparum* antigens: three soluble recombinant proteins (PF13, PfMSP1, and PfAMA1), and bovine serum albumin-conjugated peptides (PfCSP and PfGLURP) from *P. falciparum*, using procedures previously described [11, 30, 31]**.** In short, antigen-coupled beads and plasma were deposited in 96-well plates and analyzed using the Luminex-MAGPIX system and xPONENT 4.1 software. IgG levels were expressed as median fluorescence intensity (MFI). A pool of sera from malaria-immune African adults and plasma samples from malaria-naive European individuals were included in each assay as positive and negative controls, respectively. Seropositive and seronegative groups were defined from MFI values as previously described [11], using two-component Gaussian mixture model (Additional file 1: Method S1 and Fig. S1).

For the purpose of this study, only the PfAMA1 antibody response was used because: (1) this marker had similar sensitivity and specificity as a latent class antigen modeled in Steinhardt et al. using all 5 previously described antigens [11], and (2) it has been shown to act as biomarker of *P. falciparum* exposure in populations with low immunity such as young children, when previously exposed individuals acquire a long-lived component of the antibody response which increases with age [13, 14].

#### Environmental and remotely sensed data

Descriptions and resolutions of environmental, climatic, and remotely sensed data are provided in supplemental information (Additional file 1: Table S1). Briefly, temporally dynamic climatic and environmental variables were downloaded from Moderate Resolution Imaging Spectroradiometer (MODIS) satellite data for each investigated *fokontany* [32]. These included the following temperature and vegetation indices (vegetation cover proxies): (1) all 8-days Land Surface Temperature (LST) and emissivity composites; and (2) all 16-days vegetation indices composites—Normalized Difference Vegetation Index (NDVI) and Enhanced Vegetation Index (EVI) [32]. For each of these indices, values matched at one, two and three months prior to the survey date were obtained for each investigated school (within a *fokontany*) [33].

The remaining environmental variables were assumed to be static for each *fokontany* including: (1) The annual MODIS land cover type product for 2014 [34]**;** (2) elevation, measured from the shuttle radar topography mission elevation surface [35]**;** and (3) health facilities location in the study area, obtained from recently published data [27]. For land cover data, the international geosphere-biosphere programme legend and class descriptions were used [36], and 5 main classes were utilized: (a) forests, (b) woodlands, (c) grasslands or cereals, (d) wet, croplands or mosaics, and (e) others’ class grouping shrublands, wetlands, barren, build-up or water bodies.

Environmental and remotely sensed data processing were performed using standard geographic information system computational techniques (Additional file 1: Method S2). The R package {MODISTools} was used for downloading and processing of MODIS data, which provides a simplified interface between R software [37] and MODIS land product subsets [32].

### Data analyses

#### Spatial distribution and clustering of P. falciparum infection risk and cumulative exposure

Descriptive and spatial analyses of malaria infection prevalence and PfAMA1 Ab IgG seroprevalence were conducted by *fokontany*. Malaria hotspots were assessed via detection of spatial autocorrelation in the data using the local Moran’s I as an indicator of spatial heterogeneity [20]. Logit scale was used to produce more normal distributions of both malaria infection prevalence and PfAMA1 Ab seroprevalence. Empirical neighbourhood of investigated *fokontany* was defined in order to have at least one neighbour and within a maximum distance, approximately 17 km between two farthest *fokontany* (Additional file 1: Fig. S2). Then, global and local Moran’s I values were calculated using Monte Carlo simulations (n = 999) and equal row-standardized spatial weights [38] to test its significance [39]. This test can be interpreted as an indicator of local pockets of non-stationarity, or hotspots, and assesses the influence of a *fokontany* on the magnitude of the global statistic to identify “outliers” [40, 41]. Functions in the R package {spdep} were used to calculate these indices; and a threshold of p < 0.05 was chosen to identify significant spatial autocorrelation.

#### Statistical models to characterize determinants of P. falciparum infection and cumulative exposure by PfAMA1 Ab data

Both *P. falciparum* infection and exposure models were carried out at two levels: (1) at *fokontany* level, to assess the effect of mean temperature and vegetation index—lagged by one month based on univariable analyses of different lagged indices (1–3 months) association with aggregated positivity of malaria infection and exposure, respectively—elevation, percentage of land cover class and distance to health facilities (Additional file 1: Table S1); (2) and at individual-level including additional demographic and household covariates (Additional file 1: Table S2). In both exposure (PfAMA1 response positivity) models the school malaria infection prevalence was included as a potential indicator (mediator) of the seropositivity response and to assess its correlation to detect higher risk *fokontany*.

Mixed-effects binomial logistic regression analyses with two observational-level random effects—to account for within-district and -commune correlations—were used to model *P. falciparum* infection and PfAMA1 Ab positivity at fokontany- and individual*-*level [42, 43]. Univariable analyses were conducted first to explore the relationship with each of the climatic, environmental, and sociodemographic variables; all covariates which showed significant effects on *P. falciparum* infection positivity (or PfAMA1 Ab seropositivity) in univariable analyses were then included in multivariable analyses. Next, sets of candidate models were compared and ranked using multi-model selection procedures through the R package {MuMIn} according to the lowest second-order Akaike information criterion [44–46] (Additional file 1: Method S3).

Data analyses were carried out using R software v3.6.0 [37] and R package {lme4} [47].

## Results

### Individual-, household- and fokontany-level characteristics

**Fig. 2:**
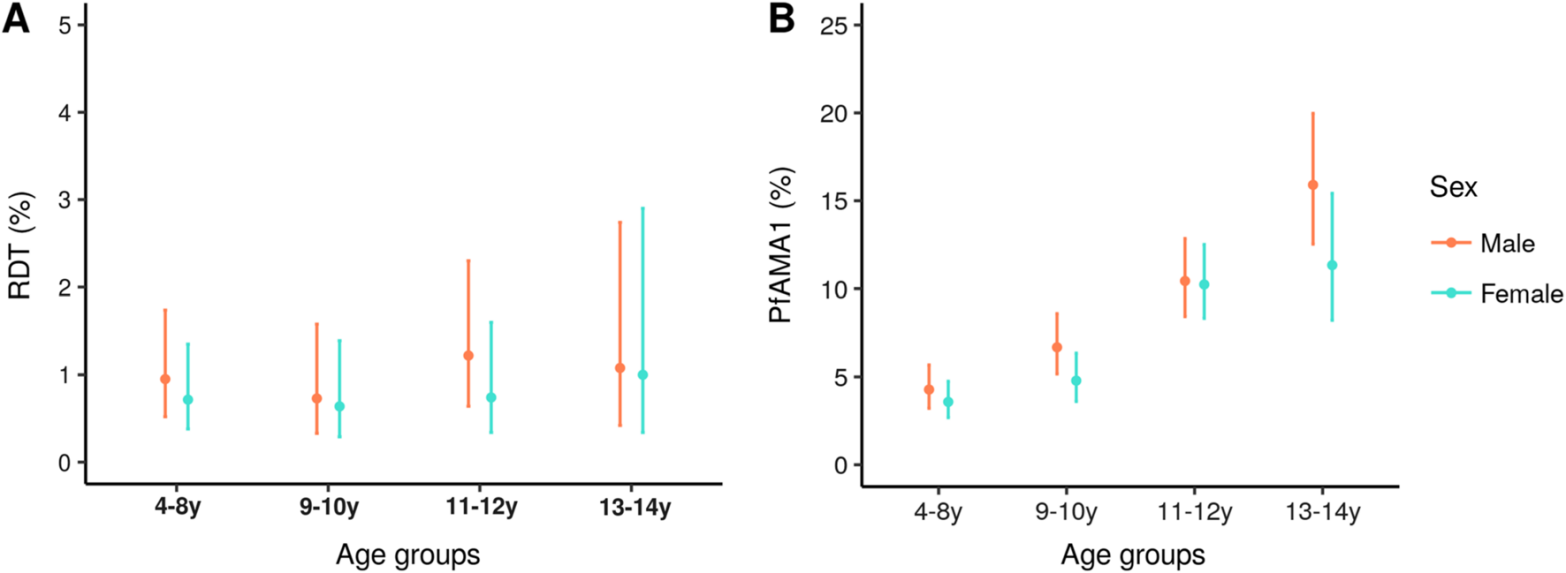
Overall distributions across age and sex of **A** malaria infection prevalence by RDT, and **B** PfAMA1 Ab seroprevalence. Vertical bars represent 95% CI of proportions

Overall, 6293 school-children ages 2–14 years were enrolled from 182 *fokontany* where investigated primary schools were located. The median age of participants was 10 years (IQR: 8–11) and 47.4% were male (2984 of 6293). About two-thirds of children sampled (64.7%, 4073 of 6293) were 2–10 years old. Most households (55.4%, 3114 of 5619) in the investigated areas had two or more ITNs for its members (median = 6.0 individuals per household, IQR = 5.0–8.0) (Table 1). A total of 53 (0.8%, 95% CI 0.6–1.1%) children tested positive for malaria by RDT, with no differences between age groups (2–8 years, 9–10  years, 11–12  years and 13–14  years) (p = 0.62). However, 443 (7.0%, 95% CI 6.4–7.7%) children were seropositive to PfAMA1 antibody with a significant increase in seropositivity across age, both for male and female (p < 0.001) (Fig. 2).

**Table 1:** Characteristics of the study participants (N = 6293), households (N = 5619) and *fokontany* (N = 182)

Fokontany-level variables	Mean (range)	Individual-level variables	n (%)
Climatic, environmental and land cover		RDT positive	53 (0.8)
NDVI lag-1	0.5 (0.3–0.8)	PfAMA1 Ab seropositive	443 (7.0)
LST Day lag-1 (°C)	25.1 (18.1–30.4)	Age (years)	
Forests (%)	1.7 (0.0–65.2)	2–8	2,310 (36.7)
Woodlands (%)	12.2 (0.0–100.0)	9–10	1,763 (28.0)
Grasslands or cereals (%)	80.0 (0.0–100.0)	11–12	1,549 (24.6)
Wet, croplands or mosaics (%)	2.6 (0.0–100.0)	13–14	671 (10.7)
Other land cover classes (%)^a^	0.2 (0.0–20.0)	Sex (male)	2,984 (47.4)
Elevation (m)	1305.3 (773.3–2140.7)	Fever last 2 weeks	481 (7.6)
		Travel in last 2 months	213 (3.4)
School RDT prevalence, % (SD)	0.9 (2.8)	Use of ITN last night	2,792 (44.4)
PfAMA1 Ab seroprevalence, % (SD)	7.2 (11.0)	Presence of RDT positive household member	24 (0.4)
Distance > 5 km from health facility, n (%)	59 (32.4)		
Household-level variables			
No. of members, mean (SD)	6.5 (2.3)		
No. of ITNs, n (%)			
0	2,148 (38.2)		
1	357 (6.4)		
2–4	2990 (53.2)		
5–10	124 (2.2)		

*NDVI* Normalized Difference Vegetation Index at the previous month; *LST* daytime Land Surface Temperature and emissivity composites at the previous month; *ITNs* insecticide-impregnated mosquito-nets; *SD* standard deviation

^a^Shrublands, wetlands, barren, or water bodies land cover

### Spatial distribution and hotspots of *P. falciparum* infection prevalence by RDT and of PfAMA1 Ab seroprevalence

**Fig. 3:**
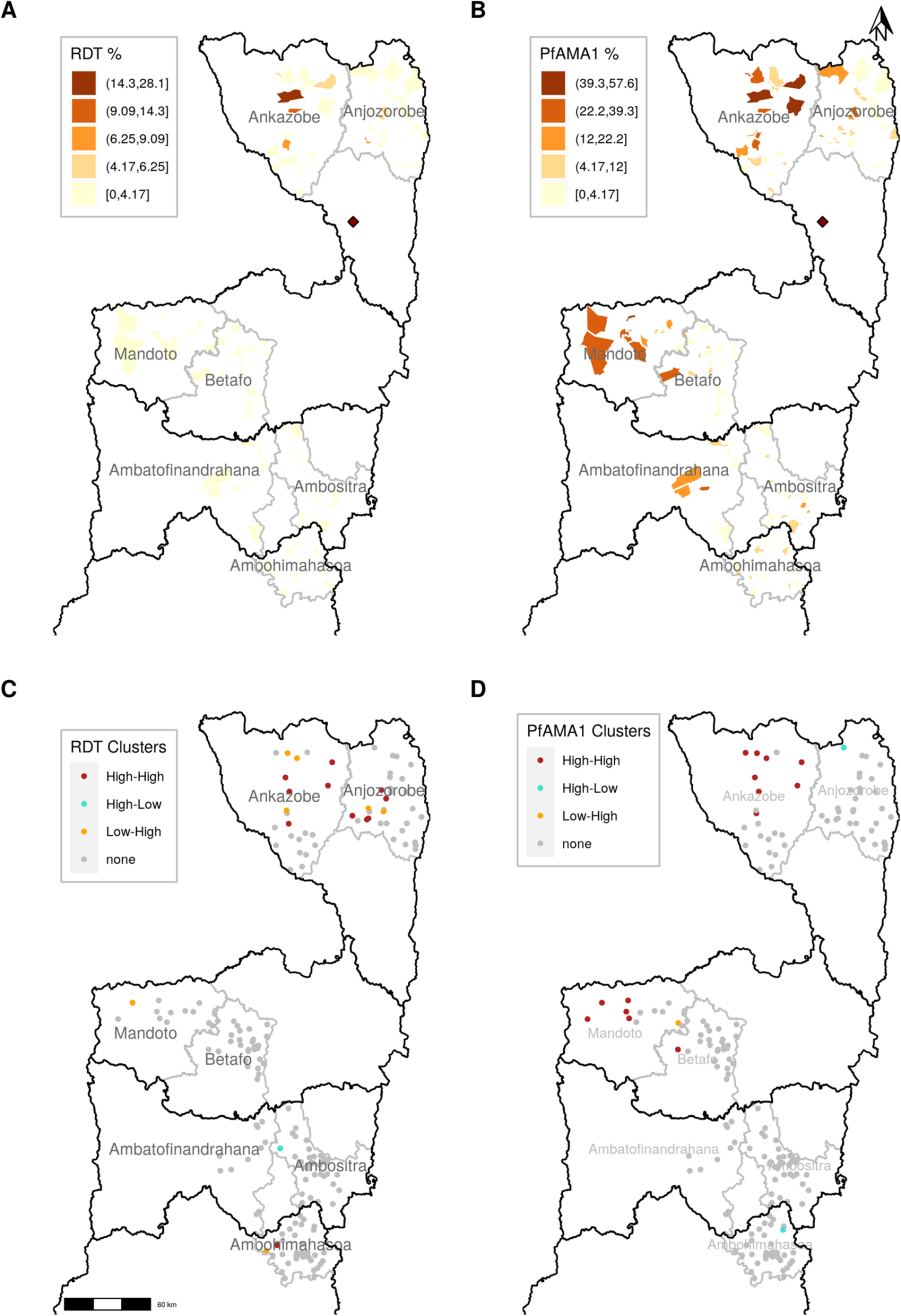
Epidemiology and local clustering of *P. falciparum* infection prevalence by RDT and PfAMA1 antibody (Ab) seroprevalence across the study area. **A** Spatial distribution of malaria infection prevalence, and **B** Spatial distribution of PfAMA1 Ab seroprevalence. Dark-maroon colored *fokontany* had higher prevalence and the optimal 5-classes by Jenks classification were used for both malaria infection by RDT and PfAMA1 Ab prevalence. The dark-red diamond represents the capital of Madagascar, Antananarivo. **C** Malaria infection prevalence clusters, and **D** PfAMA1 Ab seroprevalence clusters. “High-High” clusters represent *fokontany* with observed values matching with the weighted mean of each *fokontany*’s neighbours, which is high; “High-Low” clusters are those with abnormally observed high but expected low values; “Low–High” clusters are those with abnormally observed low but expected high values; and no deviance from the stationarity assumption are marked with “none”. These figures are supported by Additional file 1: Fig. S4 and Fig. S5, Additional file 2: Table S3 and Additional file 3: Table S4

Malaria infection by RDT was highest in the *fokontany* located in the northern districts of Ankazobe and Anjozorobe. Overall, only 32 out of 182 *fokontany* had a malaria infection prevalence greater than or equal to 1%, the highest prevalence being 28.1% (Fig. 3A). PfAMA1 Ab seroprevalence was higher than malaria infection prevalence overall (Fig. 2), both in *fokontany* in the northern and western parts of the study site, with higher heterogeneity in its distribution. For instance, PfAMA1 Ab seroprevalence was greater than 12.0% in *fokontany* of 5 different districts across the study area (Ankazobe, Anjozorobe, Mandoto, Betafo and Ambatofinandrahana) (Fig. 3B).

Spatial autocorrelation and hotspots of malaria infection prevalence and PfAMA1 Ab seroprevalence were identified across the study sites. Positive and significant Moran’s I indices were found for both *P. falciparum* infection prevalence and Ab seroprevalence, with values of 0.24 (p = 0.001) and 0.59 (p = 0.001) respectively, indicative of spatial clustering of areas with similar malaria transmission (high or low). High clusters *fokontany* are the combination of “High-High” or “Low–High” clusters; that represent *fokontany* with expected values (prevalence or seroprevalence) matching with the weighted mean of each *fokontany*’s neighbours, or those with abnormally observed low but expected high values, respectively (Fig. 3C and D). For *P. falciparum* infection prevalence, hotspots (“High-High” clusters) were identified mostly in one single area in the northern districts of Ankazobe and Anjozorobe (Fig. 3C). Overall, high clusters identified across districts were consistent using both RDT prevalence or PfAMA1 Ab seroprevalence. However, hotspots of PfAMA1 Ab seroprevalence were not observed in Anjozorobe but rather in the southern district of Mandoto (Fig. 3D). *Fokontany* with seroprevalence greater than 10% were found in Ankazobe and Mandoto (Additional file 1: Fig. S3). Thus, analyses of Abs revealed nearly twice as many hotspots as those based on *P. falciparum* infection prevalence by RDT, suggesting some level of agreement between both detection methods.

### Local determinants of *P. falciparum* infection by RDT and exposure by PfAMA1 Ab at the fokontany-level

For *P. falciparum* infection model, univariable analyses at the fokontany-level revealed significant positive associations with longer distance to health facilities, higher temperatures the previous month, and percentage of land cover classes such as grasslands or cereals. Negative associations were observed between *P. falciparum* infection and the *fokontany* at higher elevation, and greater values of NDVI during the previous month. However, the very low RDT prevalence observed in the study population (53 RDT + out of 6,293) prevented finding consistent factors associated in multivariable analyses: only higher temperatures the previous month in a *fokontany* was found as risk factor of malaria infection at fokontany-level (Table 2), and a fever episode in the previous two weeks at individual-level (Additional file 1: Table S5).

**Table 2:** *P. falciparum* infection and exposure (PfAMA1 Ab response) model mixed-effects regression models at fokontany-level

Factors associated	*P. falciparum* infection model				*P. falciparum* exposure (PfAMA1 Ab response) model			
	Univariable		Multivariable		Univariable		Multivariable	
	OR	95%CI	OR^e^	95%CI	OR	95%CI	OR^e^	95%CI
Health Facilities > 5 km	1.8	1.1–3.1			2.1	1.8–2.6	1.6	1.2–2.2***
School RDT prevalence^c^					4.4	3.5–5.6	1.9	1.2–3.1**
NDVI at lag-1^a^	0.7	0.6–1.0			0.8	0.7–0.9	1.0	0.8–1.3
LST Day at lag-1^b^	5.4	3.0–9.7	8.9	2.9–28.0***	4.5	3.7–5.5		
Grasslands or cereals^c^	1.2	1.1–1.4			1.1	1.1–1.2	0.9	0.9–1.0
Forests^c^	0.7	0.3–1.5			0.8	0.7–1.0		
Woodlands^c^	0.8	0.7–1.0	1.2	0.9–1.5	0.9	0.8–0.9		
Wet, croplands or mosaics^c^	0.8	0.5–1.3			0.9	0.8–1.0		
Elevation^d^	0.7	0.6–0.8			0.7	0.6–0.7	0.7	0.6–0.8***

Wald-test approximation was used for CIs (confidence interval) and p-values

*NDVI* Normalized Difference Vegetation Index at the previous month; *LST* daytime Land Surface Temperature and emissivity composites at the previous month; *OR* odds ratio

^a^NDVI was scaled 1/10, as one unit increase means 0.1 increase

^b^LST Day was scaled in 5 °C unit, as one unit increase means 5° C increase

^c^Variables scaled in 10% unit, as one unit increase means 10% increase

^d^Elevation scaled in 100 m, as one unit increase means 100 m increase

^e^Adjusted odds ratio

***p-value < 0.001; **p-value < 0.01; *p-value < 0.05

For *P. falciparum* exposure (PfAMA1 Ab response) model, univariable analyses at the fokontany-level revealed significant positive associations with distance to health facilities and school RDT prevalence, while associations with environmental and climatic indicators were more variable. Exposure to *P. falciparum* tended to increase with higher temperatures the previous month, and with increased percentage of grasslands or cereals land cover classes in a *fokontany*; while it decreased with higher values of vegetation (NDVI) the previous month, the percentage of woodlands, or elevation (Table 2). After excluding variables with strong collinearity (Additional file 1: Figs. S6 and S7) and adjusting for the effect of other variables in the multivariable model of exposure to *P. falciparum*, living further than 5 km from a health facility was associated with increased odds of exposure (adjusted odds ratio (OR) = 1.6, 95% CI [1.2–2.2]), and every 10% (one unit) increase in school-level RDT prevalence was associated with a doubling in the odds of PfAMA1 Ab response aggregated at fokontany-level (OR = 1.9, 95% CI [1.2–3.1]). Out of all the environmental and climatic variables, only elevation was significantly associated with *P. falciparum* exposure, with a 30% decrease in the odds for every 100 m (one unit) increase in elevation (OR = 0.7, 95% CI [0.6–0.8]) (Table 2).

No evidence of residual spatial autocorrelation was found in the final multivariable model for PfAMA1 Ab response at the fokontany-level; and no deviance to normal distribution was observed to its residuals (Table 2, Additional file 1: Figs. S8 and S9). That suggests that spatially-structured factors were accounted for in the model of *P. falciparum* exposure risk, or the considered district-and-commune level random effects.

### Factors associated with PfAMA1 antibody seropositivity at the individual-level

**Fig. 4:**
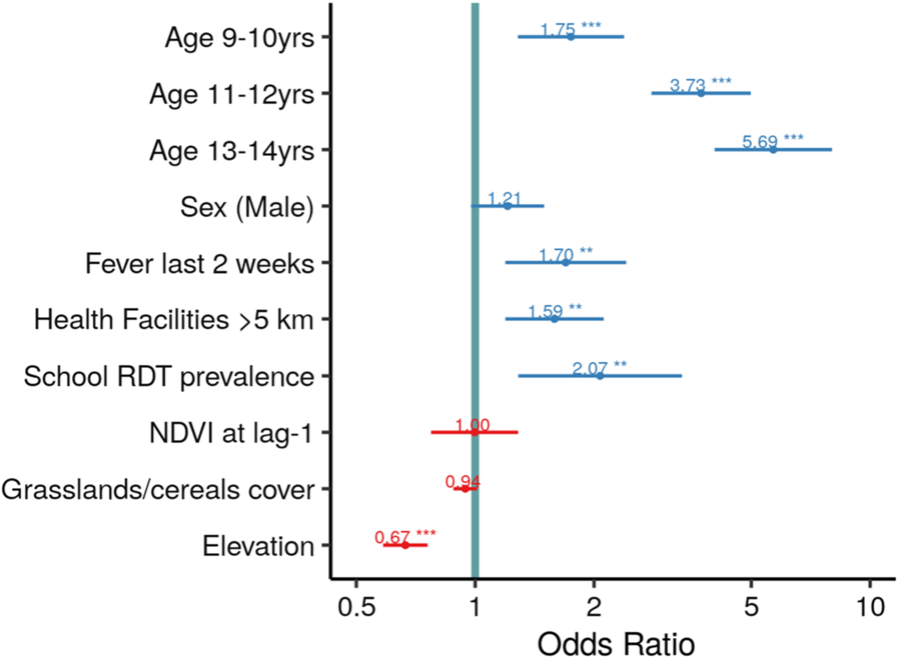
Factors associated with PfAMA1 Ab seropositivity model of individual-level covariates. Blue- and red-horizontal bars represent the 95% CI of odds ratio (ORs) of each factors with associated positive and negative effect, respectively. The green line represents the ORs value equal to one (1). This figure is supported by Additional file 1: Table S2. NDVI was scaled 1/10, as one unit increase means 0.1 increase. School RDT prevalence and grasslands/cereals cover were scaled in 10% unit, as one unit increase means 10% increase. Elevation was scaled in 100 m, as one unit increase means 100 m increase. Levels of significance are marked with (***) for p < 0.001, (**) for p < 0.01, and (*) for p < 0.05

Individual seropositivity decreased with higher values of vegetation (NDVI) the previous month, the percentage of land cover such as woodlands, and the elevation of *fokontany* (Additional file 1: Table S2). When adjusting for individual-level factors in the multivariable model, distance from health facilities, school-level RDT prevalence, and elevation were remained statistically significant, with similar coefficients as in the *P. falciparum* exposure (PfAMA1 Ab response) model at the fokontany-level (Fig. 4). In addition, the probability of being seropositive increased with age: children aged 9–10 years, 11–12  years and 13–14  years were likely to be more seropositive than youngest 2–8  years group, and the corresponding ORs [95% CI] were, respectively, 1.8 [1.3–2.4], 3.7 [2.8–5.0] and 5.7 [4.0–8.0]. Having a fever episode in the previous two weeks (OR = 1.7 [1.2–2.4]) was also identified as risk factor.

## Discussion

Pockets of residual transmission in low transmission settings can pose significant challenges to achieving malaria elimination goals [48]. Research on malaria transmission heterogeneity in settings pursuing elimination is therefore important to allow better targeting of malaria control activities. Since data collection for this study was conducted, Madagascar and other low-income countries have seen a trend of increasing malaria incidence, which further justifies the need of new approaches to reverse this trend [49]. Using a seroprevalence study of 6293 school-children in the highlands of Madagascar (CHL and Fringe areas) to characterize the spatial heterogeneity of *P. falciparum* infection, approximately 7.0% of children had been exposed to *P. falciparum* according to the Ab response despite a very low prevalence of malaria infection using RDTs (0.8%). Furthermore, hotspots of PfAMA1 Ab seroprevalence were observed in multiple districts in the area, many of which were not observed via malaria RDTs. Exposure to the malarial parasite, as indicated by seroprevalence, increased with a child’s age (Fig. 2), for children living further than 5 km from a health facility, and for those experiencing a fever episode in the previous 2 weeks.

Spatial heterogeneity of malaria infection prevalence and PfAMA1 Ab seroprevalence were confirmed across the study sites, revealing an important number of seroprevalence hotspots in districts of the northern and southern part of CHL and Fringe areas of Madagascar. These findings are in accordance with a previous serological study carried out in Madagascar [50], and similar heterogeneities in malaria transmission were found using routine surveillance data in west Fringes of highlands (Fig. 3) [3, 4]. Factors associated with these spatial heterogeneities were further characterized at the fokontany-level. In this study, two factors associated with *P. falciparum* cumulative exposure at the *fokontany* and individual levels were school *P. falciparum* infection prevalence by RDT and having a fever in the previous 2 weeks, respectively. This suggests, on one hand, that PfAMA1 Ab may capture additional information on cumulative exposure to malaria parasite in children helping to identify a greater proportion of transmission hotspots; on the other hand, the correlation between RDT and PfAMA1 suggests that conventional RDTs can still be used in settings pursuing elimination, especially in areas with high-density infections [23, 24] not able to afford the additional operational costs of serological studies. Further studies are needed to better understand the cost-effectiveness of more accurate diagnostic techniques for low-density infections such as PCR [51].

Children with geographical barriers in health care access, who lived further than 5 km from health facilities (around one third of the study population) were significantly more exposed to *P. falciparum*, which could have important implications for malaria elimination efforts in these areas. These results suggest that suboptimal testing and treatment of malaria infections in these areas, due to geographic barriers to access health care [52], could result in undetected pockets of malaria transmission that undermine elimination goals. Indeed, previous studies have shown that persistent geographic inequalities in health care access still exist in rural areas of Madagascar, with an exponential decrease in the use of health facilities over the first 5 km [53]. Community health programs can be an effective way to remove geographic barriers to health care access [54], since two community health workers are present in every *fokontany* independently of their distance to a health facility. However, current national guidelines for community case management of malaria only target children under 5 years, the group at the highest risk of mortality from malaria infections. In this study, this group was found to be least exposed to *P. falciparum* (with zero seropositive to PfAMA1 Ab) although it was not representative of this age group because the study was restricted to school-aged children; and in rural Madagascar, children generally start school at age 5. A sub-analysis of factors associated with exposure of ≤ 5 years children (seroprevalence = 2.6% [5 of 194]) suggests the absence of the barrier of distance to health facilities (Additional file 1: Table S6), which may indicate effective case management but could also be due to small sample sizes for this group. In parallel, this national strategy of malaria community case management leaves out the vast majority of the population, and current plans to expand it to all ages in Madagascar [55] could, therefore, help address symptomatic malaria cases in these pockets of malaria transmission and accelerate elimination efforts.

Among environmental factors associated with *P. falciparum* cumulative exposure, PfAMA1 Ab response at the fokontany-level was higher at lower elevations, but there was little additional effect of other environmental and climatic factors examined. Elevation is widely used in malaria mapping as an established proxy of malaria transmission due to its association with precipitation and temperature which, along with vegetation cover, are generally found to be important predictors of malaria incidence and transmission, given their role on *Anopheles* spp survival, development, breeding, and biting rates [56–60]**.** In this setting, elevation was significantly and negatively correlated with temperature and grasslands or cereals land covers, but positively with vegetation cover proxies (NDVI and EVI) (Additional file 1: Fig. S6 and Fig. S7), which might explain why these variables had little to no effect in the final multivariable models (Table 2, Fig. 4).

At the individual level, *P. falciparum* exposure risk increased with age, which could be due to repeated exposure of children—acquiring a long-lived component of the Ab response—to infective female *Anopheles* mosquito bites. Antibody responses are boosted by active *P. falciparum* infections as children get older, which is similar to endemic areas and informative for characterizing spatial heterogeneity [14]. In addition, behaviour and access to protective measures vary for different age groups: given the targeting of children under 5 years and pregnant women in malaria control strategies, net use is especially important in older children and adolescents, but tends to be lowest in these age groups [61–63]. In this setting, 56.4% of children were in households with two or more bed nets, but neither the individual use of an ITN nor the number of ITNs in a household was associated with PfAMA1 Ab seropositivity. Moreover, other studies have found that older children spent more time outdoors in the evening, when *Anopheles* spp biting rates are typically higher, putting them at higher risk for being bitten by infective mosquito than other age groups [64, 65]. That might explain the important role played by these older children and adolescents as reservoirs that could sustain malaria transmission [6], even in very low transmission risk settings of Madagascar (< 1% parasite prevalence) as previously highlighted by Kang et al*.* [33].

This study had several limitations**.** First, the cross-sectional design reflects a snapshot of children’s infection or cumulative exposure, dependent on underlying study setting contexts. Further, these findings might not be representative of other low malaria transmission settings in the highlands of Madagascar due to their diversity in malaria transmission dynamics (central, fringe east versus fringe west highlands) and vector ecology [26, 66]. Second, since data in this study were collected in 2014, a more recent serological survey could give better information on malaria transmission intensity, to inform how Madagascar should adapt its interventions to reverse the current trend of increasing malaria cases [49].

## Conclusions

In this setting, serological markers (PfAMA1 Ab) enabled to highlight hotspots of malaria seroprevalence in multiple districts in the highlands of Madagascar (CHL and Fringe areas)—many of which were not observed via malaria RDTs—and associated factors, revealing a pattern of higher transmission linked to geographical barriers in health care access. Targeting these residual pockets could reduce malaria transmission at the community level [67, 68]**.** Nevertheless, sub-optimal testing and treatment of malaria infections in CHL and surrounding Fringes areas could undermine elimination efforts by NMCP, at the moment when Madagascar should adapt interventions to face the current challenge of plateauing or increasing malaria cases [5, 49]. Serological markers—especially when used in young children—could add benefits to routine malaria surveillance, provide a good picture of malaria transmission structure [13], and help to support and evaluate community interventions aimed at elimination [15].

## Disclaimer

The findings and conclusions in this report are those of the author(s) and do not necessarily represent the official position of the Centers for Disease Control and Prevention.

## Supplementary Information

**Additional file 1: Method S1.** Two-component gaussian mixture model; **Method S2.** Environmental and remotely sensed data processing; **Method S3.** Mixed-effects binomial logistic regression model frameworks and selection; **Table S1.** List of environmental and climatic data; **Table S2.** Univariable and multivariable *P. falciparum* exposure (PfAMA1 Ab response) model of individual-, household- and fokontany-level covariates, using mixed-effect logistic regression at district and commune level; **Figure S1.** Cut-off value for PfAMA1 Ab seropositivity (dashed-red line) by using two finite Gaussian mixture models and the serological data (for children and adult participants, n = 12,770) described in Steinhardt et al. [11]*;* **Figure S2.** *Fokontany* neighbour definition by maximum distance, using Great Circle distance around 17 km between two contiguous *fokontany*; **Figure S3.** Malaria infection prevalence by RDT versus PfAMA1 Ab seroprevalence detected high clusters *fokontany* across districts. High clusters *fokontany* are the combination of “High-High” or “Low–High” clusters; that represent *fokontany* with expected values (prevalence or seroprevalence) matching with the weighted mean of each *fokontany*’s neighbours, or those with abnormally high expected but low expected values, respectively; **Figure S4.** Weighted global Moran’I statistics versus simulated random distribution of logit scale of (A) malaria infection prevalence by RDT (I = 0.24), and (B) PfAMA1 Ab seroprevalence (I = 0.59); **Figure S5.** Moran Scatterplots of clusters and hotspots of (A) malaria infection prevalence by RDT, and (B) PfAMA1 Ab seroprevalence; **Figure S6.** Pearson’s correlation scatterplot and peer’s significance of *P. falciparum* infection prevalence by RDT, PfAMA1 Ab seroprevalence and quantitative environmental and climatic covariates. Levels of significance are marked with (***) for p < 0.001, (**) for p < 0.01, and (*) for p < 0.05. Lagged values of temperature and vegetation at 2 and 3 months were less correlated to malaria infection prevalence by RDT and PfAMA1 Ab seroprevalence than one-month lag at fokontany-level; **Figure S7.** Pearson’s correlation scatterplot and peers of *P. falciparum* infection prevalence by RDT, PfAMA1 Ab seroprevalence and quantitative environmental and climatic covariates associations. There was high correlation between multiple pairs of covariates such as {NDVI, EVI, forests, woodlands, or grasslands/cereals}, {LST day, LST night, elevation, woodlands or grasslands/cereals} and {grasslands/cereals, wet/croplands/mosaics}; **Table S5.** Univariable and multivariable *P. falciparum* infection model at individual-, household- and fokontany-level covariates using mixed-effect logistic regression at district and commune level; **Figure S8.** *P. falciparum* exposure (PfAMA1 Ab response) model residuals diagnostics of normality at fokontany-level. No significant deviance to normal distribution was observed in PfAMA1 Ab seroprevalence model residuals using Kolmogorov–Smirnov normality test (p = 0.3); **Figure S9.** Spatial autocorrelation of residuals diagnostics using Moran’s I index of non-spatial *P. falciparum* exposure (PfAMA1 Ab response) model at fokontany-level; **Table S6.** Sub-model for $\le$ 5 years old children (seroprevalence = 2.6% [5 of 194]), only variables included in the all participants Ab response model were included: univariable *P. falciparum* exposure (PfAMA1 Ab response) model of individual- and fokontany-level covariates, using binomial logistic regression.

**Additional file 2: Table S3.** Malaria infection prevalence by RDT measures of local indicators of spatial association.

**Additional file 3: Table S4.** PfAMA1 Ab seroprevalence measures of local indicators of spatial association.

## Data Availability

The datasets used and/or analysed during the present study are available from the corresponding author upon reasonable request.

## Abbreviations

*P.** falciparum*: *Plasmodium* *falciparum*
PfAMA1: *Plasmodium falciparum* Apical membrane antigen 1
Ab: Antibody
RDTs: Rapid diagnostic tests
NMCP: National Malaria Control Program
IgG: Immunoglobulin G
ITNs: Insecticide-treated mosquito nets
IRS: Indoor residual spraying
CHL: Central Highlands of Madagascar
CI: Confidence interval
PCR: Polymerase chain reaction
MODIS: Moderate resolution imaging spectroradiometer
LST: Land Surface Temperature and emissivity composites
NDVI: Normalized Difference Vegetation Index
EVI: Enhanced Vegetation Index
OR: Odds ratios
IQR: Inter-quartile range
