# Supplementary material for: Identification of factors associated with residual malaria transmission using school-based serological surveys in settings pursuing elimination
Source: Malar J. 2022 Aug 21;21:242. doi: 10.1186/s12936-022-04260-0 (PMC9392911; doi:10.1186/s12936-022-04260-0)
Supplement: Supplementary file 1 — Additional file 1: Method S1. Two-component gaussian mixture model; Method S2. Environmental and remotely sensed data processing; Method S3. Mixed-effects binomial logistic regression model frameworks and selection; Table S1. List of environmental and climatic data; Table S2. Univariable and multivariable P. falciparum exposure (PfAMA1 Ab response) model of individual-, household- and fokontany-level covariates, using mixed-effect logistic regression at district and commune level; Figure S1. Cut-off value for PfAMA1 Ab seropositivity (dashed-red line) by using two finite Gaussian mixture models and the serological data (for children and adult participants, n = 12,770) described in Steinhardt et al. [11]; Figure S2. Fokontany neighbour definition by maximum distance, using Great Circle distance around 17 km between two contiguous fokontany; Figure S3. Malaria infection prevalence by RDT versus PfAMA1 Ab seroprevalence detected high clusters fokontany across districts. High clusters fokontany are the combination of “High-High” or “Low–High” clusters; that represent fokontany with expected values (prevalence or seroprevalence) matching with the weighted mean of each fokontany’s neighbours, or those with abnormally high expected but low expected values, respectively; Figure S4. Weighted global Moran’I statistics versus simulated random distribution of logit scale of (A) malaria infection prevalence by RDT (I = 0.24), and (B) PfAMA1 Ab seroprevalence (I = 0.59); Figure S5. Moran Scatterplots of clusters and hotspots of (A) malaria infection prevalence by RDT, and (B) PfAMA1 Ab seroprevalence; Figure S6. Pearson’s correlation scatterplot and peer’s significance of P. falciparum infection prevalence by RDT, PfAMA1 Ab seroprevalence and quantitative environmental and climatic covariates. Levels of significance are marked with (***) for p < 0.001, (**) for p < 0.01, and (*) for p < 0.05. Lagged values of temperature and vegetation at 2 and 3 months were less correlated to ma [file 12936_2022_4260_MOESM1_ESM.docx]

**Identification of factors associated with residual malaria transmission using school-based serological surveys in settings pursuing elimination**

Jean Marius Rakotondramanga^1,2,3,4*^, Inès Vigan-Womas^5,6^, Laura C. Steinhardt^7^, Aina Harimanana^1^, Elisabeth Ravaoarisoa^8^, Tsikiniaina L Rasoloharimanana^6^, Seheno Razanatsiorimalala^9^, Amy Wesolowski^10^, Milijaona Randrianarivelojosia^9,11^, Benjamin Roche^2,4¶^, Andres Garchitorena^1,4¶^

**Author details**

^1^ Epidemiology and Clinical Research Unit, Institut Pasteur de Madagascar, Antananarivo, Madagascar.

^2^ IRD, Sorbonne Université, UMMISCO, Bondy F-93143, France.

^3^ Sorbonne Université, ED 393, Paris, France

^4^ MIVEGEC, Univ. Montpellier, CNRS, IRD, Montpellier, France.

^5^ Immunology of Infectious Diseases Unit, Institut Pasteur de Madagascar, Antananarivo, Madagascar.

^6^ Institut Pasteur de Dakar, Immunophysiopathology and Infectious Diseases Department, Dakar, Senegal.

^7^ Malaria Branch, Division of Parasitic Diseases and Malaria, Center for Global Health, Centers for Disease Control and Prevention, Atlanta, Georgia, USA.

^8^ Faculty of Sciences, University of Antananarivo, Antananarivo, Madagascar.

^9^ Malaria Research Unit, Institut Pasteur de Madagascar, Antananarivo, Madagascar.

^10^ Department of Epidemiology, Johns Hopkins Bloomberg School of Public Health, Baltimore, MD, USA.

^11^ Faculté des Sciences, Université de Toliara, 601, Toliara, Madagascar.

^*^Corresponding author

Email: [jean.marius.g@gmail.com](mailto:jean.marius.g@gmail.com)

^¶^ These authors share senior authorship

**Method S1** Two-component gaussian mixture model

To dichotomize seropositivity, log-transformed of median fluorescence intensity (MFI) values were assumed to have two underlying components for seronegative and seropositive groups [1]. Two finite mixture models with a Gaussian distribution were fitted by maximum likelihood estimation using expectation-maximum algorithm with the R package{flexmix}. This statistical approach has been used on samples from other low-endemicity settings without requiring antibody responses from a reference population [2–4]. BSA (GeneCust) described in and based on Steinhardt et al. was used as carrier control. Due to skewness in all participants’ data distribution, negative values of {MFI minus BSA-background} were recoded to 1 to accommodate to the log transformation (to have more normal distribution); then all participants could be included in the study [3, 4]**.** A cut-off value for seropositivity, the junction of lower and upper Gaussian distributions, was determined to characterize seronegative (unexposed) and seropositive (exposed) populations—instead of using the commonly used mean + 3*SD cut-off (dashed-orange line) which requires an appropriate unexposed reference population (**Fig. S1**).

**
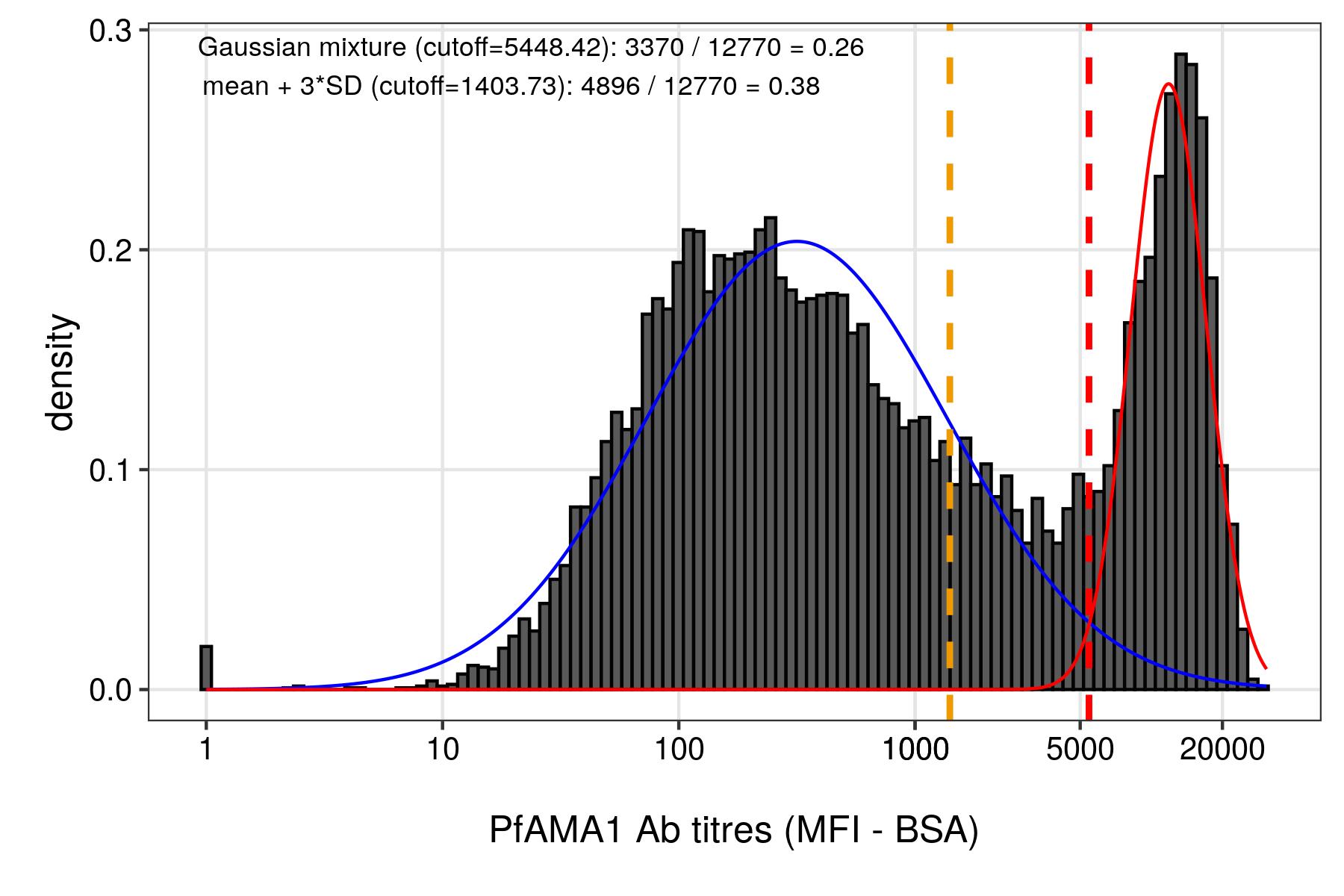
**

**Fig. S1**  Cut-off value for PfAMA1 Ab seropositivity (dashed-red line) by using two finite Gaussian mixture models and the serological data (for children and adult participants, n = 12,770) described in Steinhardt et al. [4].

**Table S1**  List of environmental and climatic data

| **Data** | **Description** | **Dynamic in models** | **Round 1 selected for *Pf.* infection and exposure (PfAMA1 Ab response) model** | **Resolution (Unit)** | **Source** |
| --- | --- | --- | --- | --- | --- |
| Distance >5 km from health facility | Calculated using Euclidean distance (>5 km, nearly 5 km in travel distance) from each fokontany centroid to the nearest health facility in QGIS | Static | Yes | Binary (km) | WHO recently published data [5, 6] |
| Elevation | Measured from the SRTM elevation surface | Static | Yes | 90 m | SRTM derivative [7] |
| NDVI | NDVI (Normalized Difference Vegetation Index) | Monthly^Ϯ^ | Lagged by 1 month prior surveys | 16-days at 250 m | MODIS product of MOD13Q1 [8, 9] |
| EVI | EVI (Enhanced Vegetation Index) | Monthly^Ϯ^ | No | 16-days at 250 m | MODIS product of MOD13Q1 [8, 9] |
| QA for NVDI or EVI* | Used for NDVI or EVI quality assessment reliability | Monthly^Ϯ^ | No | 16-days at 250 m | MODIS product of MOD13Q1 [8, 9] |
| LST Day | Day-time Land Surface Temperature (LST) and emissivity composites | Monthly^Ϯ^ | Lagged by 1 month prior surveys | 8-days at 1 km | MODIS product of MOD11A2 [8, 9] |
| LST Night | Night-time LST and emissivity composites | Monthly^Ϯ^ | No | 8-days at 1 km | MODIS product of MOD11A2 [8, 9] |
| QA for LST Day* | Used for LST Day quality assessment reliability | Monthly^Ϯ^ | No | 8-days at 1 km | MODIS product of MOD11A2 [8, 9] |
| QA for LST Night* | Used for LST Night quality assessment reliability | Monthly^Ϯ^ | No | 8-days at 1 km | MODIS product of MOD11A2 [8, 9] |
| Forests | IGBP forests legend and class | Annual^¶^ | Yes | Annual^¶^ at 500 m | MODIS product of MCD12Q1 [10] |
| Woodlands | IGBP woodlands legend and class | Annual^¶^ | Yes | Annual^¶^ at 500 m | MODIS product of MCD12Q1 [10] |
| Grasslands/cereals | IGBP grasslands or cereals legend and class | Annual^¶^ | Yes | Annual^¶^ at 500 m | MODIS product of MCD12Q1 [10] |
| Wet/croplands/mosaics | IGBP wet, croplands or mosaics legend and class | Annual^¶^ | Yes | Annual^¶^ at 500 m | MODIS product of MCD12Q1 [10] |
| Others' land cover classes | Others IGBP legends and class | Annual^¶^ | No | Annual^¶^ at 500 m | MODIS product of MCD12Q1 [10] |
| QGIS: QGIS Team Development Open Source Geospatial Foundation Project ; SRTM: SRTM Shuttle Radar Topography Mission ; MODIS: Moderate Resolution Imaging Spectroradiometer ; *QA: Quality Assessment reliability index ; IGBP: International Geosphere-Biosphere Programme ; ^Ϯ^One, two and three month prior survey ; ^¶^2014 | | | | | |

**Method S2**  Environmental and remotely sensed data processing

Temporally dynamic climatic and environmental, (1) all 8-days Land Surface Temperature (LST) and emissivity composites (day-time and night-time LST products of MOD11A2 band at 1 km spatial resolution), and (2) all 16-days vegetation indices composites (Normalized Difference Vegetation Index (NDVI) and Enhanced Vegetation Index (EVI) of MOD13Q1 band at 250 m spatial resolution) [8], indices’ values were first rasterized. Then, they were masked with corresponding quality assessment reliability composites to set and unsure “nodata” removing (such as due to cloud cover), and cropped with the fokontany boundary (only the pixels with majority surface superimpose the fokontany polygons were considered). For computational use, all pixel values at specific dates—depending on the temporal data resolution: 3 or 4 dates for temperatures, and 2 dates per month for vegetation indices—of each indices were aggregated to obtain median value at fokontany-level; and mean of monthly values from multiple raster images were calculated.

The MODIS land cover type product (MCD12Q1, at annual and 500 m spatial resolution) for 2014: (a) forests, (b) woodlands, (c) grasslands or cereals, (d) croplands or mosaics, and (e) others’ class grouping shrublands, wetlands, barren or water bodies were processed differently. The land cover class values were first rasterized and cropped with the fokontany boundary (only the pixels with majority surface superimpose the fokontany polygons were considered). Then, the percentage of fokontany *i,* area occupied by land cover class *j,* were calculated using (Eq. 1):

| $\left\{ Land cover \right\}_{i}\left( \% \right)= \frac{100 * \left\{ Number of pixels \right\}_{ij}}{\left\{ Number of all pixels \right\}_{i}}$ | (Eq. 1) |
| --- | --- |

For elevation data, mean values per fokontany were used.

QGIS software v3.10, was used to calculate the Euclidean distance from each fokontany centroid to the nearest health facility via use of World Health Organization localization data [5, 6]**.**


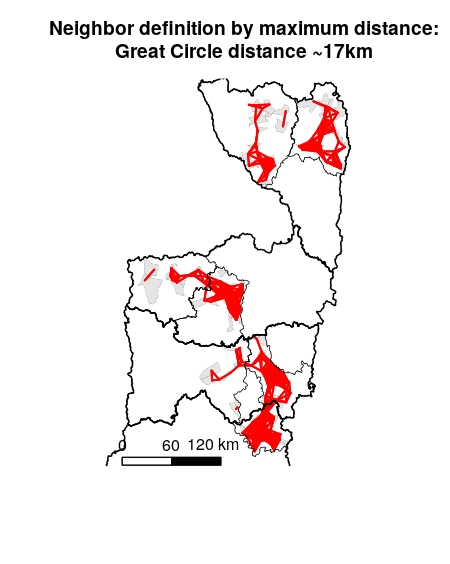


**Fig. S2**  Fokontany neighbour definition by maximum distance, using Great Circle distance around 17 km between two contiguous fokontany.

**Method S3**  Mixed-effects binomial logistic regression model frameworks and selection

Mixed-effects binomial logistic regression models with district- and commune-level random effects were fitted to model *P. falciparum* infection at fokontany-level (Eq. 2) or at individual-level (Eq. 3), and PfAMA1 Ab response at fokontany-level (Eq. 4) or seropositivity at individual-level (Eq. 5) [11, 12], using R package {lme4} [13]:

Random effects were treated as nested for the analyses to model the overdispersion and give more accurate estimates of standard errors [11, 12], because each fokontany (n = 182) or individual (n = 6,293) was surveyed only within one commune (n = 93) and one district (n = 7):

1. *P. falciparum* infection model at fokontany-level :

| $logit(pi)=\alpha_{ijk}+\beta_{0}+\beta_{1}\{distance >5 km\}+\beta_{2}\{forests\}+\beta_{3}\{woodlands\}+\beta_{4}\{grasslands/cereals\}+\beta_{5}\{wet/croplands/mosaics\}+\beta_{6}\{elevation\}+\beta_{7}\{NDVI, lag-1\}+\beta_{8}\{LST, lag-1\}+\beta_{9}\{(NDVI, lag-1)*(LST, lag-1)\}+\beta_{10}\{(NDVI, lag-1)*(grasslands/cereals)\}+\epsilon_{i} ;$ | (Eq. 2) |
| --- | --- |

where *p, i, j* and *k* are respectively the aggregated *P. falciparum* infection, the *fokontany*-, commune- and district-indices;

$\epsilon_{i}$ *~ Normal(0,* $\text{σ}_{\epsilon}^{2}$*).*

1. *P. falciparum* infection model at individual-level :

| $logit(pi)=\alpha_{ijk}+\beta_{0}+\beta_{1}\{age group\}+\beta_{2}\{sex\}+\beta_{3}\{fiver \}+\beta_{4}\{trip\}+\beta_{5}\{net use\}+\beta_{6}\{net number\}+\beta_{8}\{distance >5 km\}+\beta_{9}\{forests\}+\beta_{10}\{woodlands\}+\beta_{11}\{grasslands/cereals\}+\beta_{12}\{wet/croplands/mosaics\}+\beta_{13}\{elevation\}+\beta_{14}\{NDVI, lag-1\}+\beta_{15}\{LST, lag-1\}+\beta_{16}\{(NDVI, lag-1)*(LST, lag-1)\}+\beta_{17}\{(NDVI, lag-1)*(grasslands/cereals)\}+\epsilon_{i} ;$ | (Eq. 3) |
| --- | --- |

where *p, i, j* and *k* are respectively the *P. falciparum* infection positivity, the individual-, commune- and district-indices;

$\epsilon_{i}$ *~ Normal(0,* $\text{σ}_{\epsilon}^{2}$*)*.

1. *P. falciparum* exposure (PfAMA1 Ab response) model at fokontany-level :

| $logit(seroposi)=\alpha_{ijk}+\beta_{0}+\beta_{1}\{distance >5 km\}+\beta_{2}\{forests\}+\beta_{3}\{woodlands\}+\beta_{4}\{grasslands/cereals\}+\beta_{5}\{wet/croplands/mosaics\}+\beta_{6}\{elevation\}+\beta_{7}\{NDVI, lag-1\}+\beta_{8}\{LST, lag-1\}+\beta_{9}\{(NDVI, lag-1)*(LST, lag-1)\}+\beta_{10}\{(NDVI, lag-1)*(grasslands/cereals)\}+\beta_{11}\{pi\}+\epsilon_{i} ;$ | (Eq. 4) |
| --- | --- |

where *p, seropos, i, j* and *k* are respectively the School RDT prevalence, the aggregated PfAMA1 Ab response, the *fokontany*-, commune- and district-indices;

$\epsilon_{i}$ *~ Normal(0,* $\text{σ}_{\epsilon}^{2}$*).*

1. *P. falciparum* exposure (PfAMA1 Ab response) model at individual-level :

| $logit\left( seroposi \right)=\alpha_{ijk}+\beta_{0}+\beta_{1}\left\{ age group \right\}+\beta_{2}\left\{ sex \right\}+\beta_{3}\left\{ fiver \right\}+\beta_{4}\left\{ trip \right\}+\beta_{5}\left\{ net use \right\}+\beta_{6}\left\{ net number \right\}+\beta_{7}\left\{ RDT positive in household \right\}+\beta_{8}\left\{ distance >5 km \right\}+\beta_{9}\{forests\}+\beta_{10}\{woodlands\}+\beta_{11}\{grasslands/cereals\}+\beta_{12}\{wet/croplands/mosaics\}+\beta_{13}\{elevation\}+\beta_{14}\{NDVI, lag-1\}+\beta_{15}\{LST, lag-1\} + \beta_{16}\{(NDVI, lag-1)*(LST, lag-1)\}+\beta_{17}\{(NDVI, lag-1)*(grasslands/cereals)\}+\beta_{18}\{pi\}+\epsilon_{i} ;$ | (Eq. 5) |
| --- | --- |

where *p, seropos, i, j* and *k* are respectively the School RDT prevalence, the PfAMA1 Ab response, the individual-, commune- and district-indices;

$\epsilon_{i}$ *~ Normal(0,* $\text{σ}_{\epsilon}^{2}$*)*.

Then, the following inclusion rules and multi-model selection procedures were applied using R package {MuMIn} [14, 15]: (a) all covariates which showed significant effects on outcome variables (*P. falciparum* infection and exposure (PfAMA1 Ab response), both at *fokontany*- and individual-level) in univariable analyses were then included in multivariable analyses; (b) one of correlated pairs of quantitative environmental and climatic covariates was excluded from the same model candidate to avoid multicollinearity by using internal arguments (subset) of *MuMIn::pdredge* function {NDVI exclusive-or woodlands}, {temperature exclusive-or grasslands/cereals}, {temperature exclusive-or elevation}, and {woodlands exclusive-or grasslands/cereals} (Additional file 1: **Fig. S7**); (c) the interaction terms {NDVI * temperature} or {NDVI * “grasslands/cereals land cover”} were also included to all model candidates to assess its effects; and (d) sets of candidate models—for *P. falciparum* infection at fokontany-level (Eq. 2) and individual-level (Eq. 3), or for PfAMA1 Ab response at fokontany-level (Eq. 4) and individual-level (Eq. 5)—were then compared and ranked one-by-one according to the lowest information criteria (AICc, Second-order Akaike Information Criterion) to provide an absolute value for the model fit [14–16].

Fixed-effect coefficients were transformed to obtain odds ratios {*OR =exp(β)*}—exponentiated model coefficients which reflect the ratio of change in outcome variable (P. *falciparum* infection or exposure) associated with each explanatory variable increase—in order to facilitate interpretation of results according the appropriate used scales.

The final model of *P. falciparum* exposure (PfAMA1 Ab response) at fokontany-level was checked for unaccounted spatial autocorrelation in the model residuals using Moran’s I index.

**References**

1. Rogier E, Wiegand R, Moss D, et al. Multiple comparisons analysis of serological data from an area of low Plasmodium falciparum transmission. Malar J. 2015. https://doi.org/10.1186/s12936-015-0955-1.

2. Dempster AP, Laird NM, Rubin DB. Maximum Likelihood from Incomplete Data Via the EM Algorithm. J R Stat Soc Ser B. 1977;39:1–22.

3. Steinhardt LC, Ravaoarisoa E, Wiegand R, Harimanana A, Hedje J, Cotte AH, et al. School-based serosurveys to assess the validity of using routine health facility data to target malaria interventions in the Central Highlands of Madagascar. J Infect Dis. 2021;223:995–1004.

4. Maina J, Ouma PO, Macharia PM, Alegana VA, Mitto B, Fall IS, et al. A spatial database of health facilities managed by the public health sector in sub Saharan Africa. Sci Data. 2019;6:134.

5. QGIS Team Development Open Source Geospatial Foundation Project. http://qgis.osgeo.org/en/site/.

6. Farr TG, Rosen PA, Caro E, Crippen R, Duren R, Hensley S, et al. The shuttle radar topography mission. Rev Geophys. 2007;45:2004

7. Oak Ridge National Laboratory Distributed Active Archive Center (ORNL DAAC). MODIS and VIIRS Land Product Subsets RESTful Web Service. 2018. https://doi.org/10.3334/ORNLDAAC/1600.

8. Kang SY, Battle KE, Gibson HS, Ratsimbasoa A, Randrianarivelojosia M, Ramboarina S, et al. Spatio-temporal mapping of Madagascar’s Malaria Indicator Survey results to assess Plasmodium falciparum endemicity trends between 2011 and 2016. BMC Med. 2018;16:71.

9. Friedl MA, Sulla-Menashe D, Tan B, Schneider A, Ramankutty N, Sibley A, et al. MODIS Collection 5 global land cover: algorithm refinements and characterization of new datasets. Remote Sens Environ. 2010;114:168–82.

10. Mauny F, Viel JF, Handschumacher P, Sellin B. Multilevel modelling and malaria: a new method for an old disease. Int J Epidemiol. 2004;33:1337–44.

11. Harrison XA. A comparison of observation-level randomeffect and Beta-Binomial models for modelling overdispersion in Binomial data in ecology & evolution. PeerJ. 2015;2015:e1114.

12. Bates D, Mächler M, Bolker BM, Walker SC. Fitting linear mixed-effects models using lme4. J Stat Softw. 2015;67:i01.

13. Bartoń K. MuMIn: Multi-model inference, software. 2020. https://cran.r-project.org/package=MuMIn. Accessed 01 Mar 2022.

14. Burnham KP, Anderson DR. Model Selection and Multimodel Inference, 2nd ed. New York: Springer; 2004. https://doi.org/10.1007/b97636.

15. Nakagawa S, Schielzeth H. A general and simple method for obtaining R2 from generalized linear mixed-effects models. Methods Ecol Evol. 2013;4:133–42.


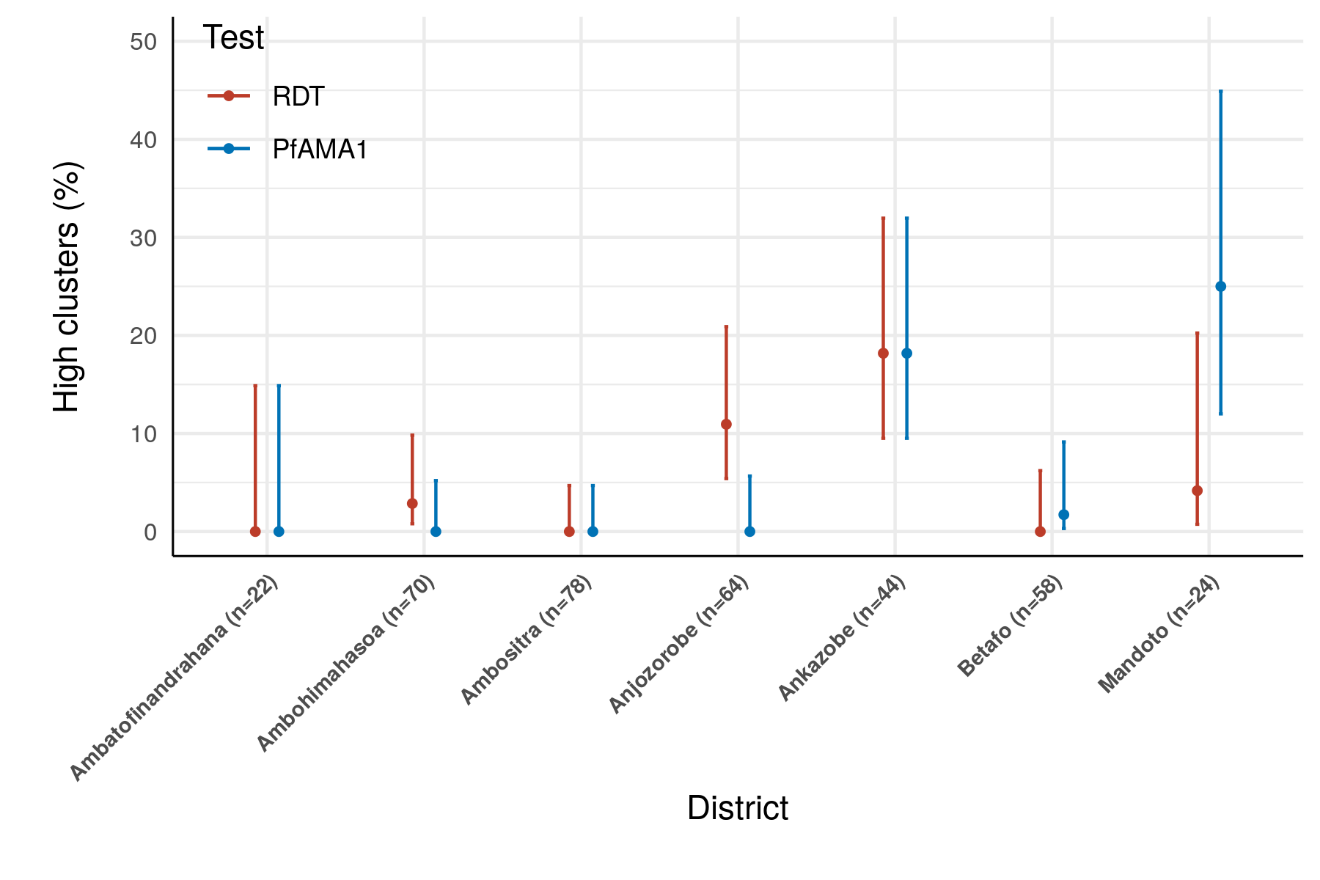


**Fig. S3** Malaria infection prevalence by RDT versus PfAMA1 Ab seroprevalence detected high clusters fokontany across districts. High clusters fokontany are the combination of “High-High” or “Low-High” clusters; that represent fokontany with expected values (prevalence or seroprevalence) matching with the weighted mean of each fokontany’s neighbours, or those with abnormally high expected but low expected values, respectively.


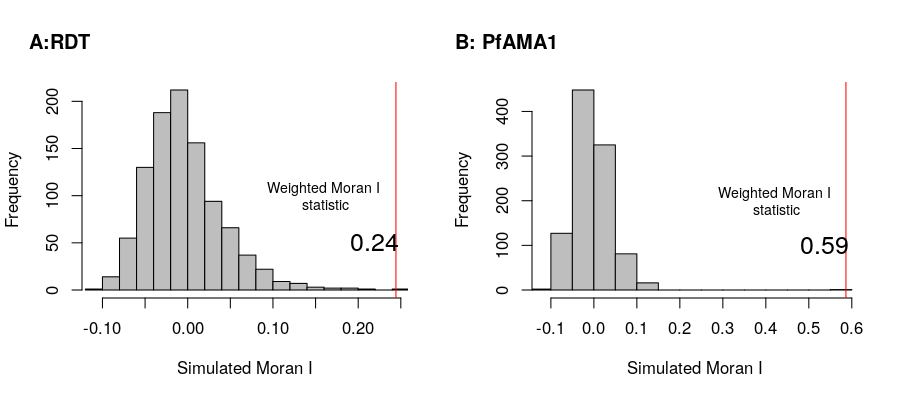


**Fig. S4** Weighted global Moran’I statistics versus simulated random distribution of logit scale of (A) malaria infection prevalence by RDT (I = 0.24), and (B) PfAMA1 Ab seroprevalence (I = 0.59)


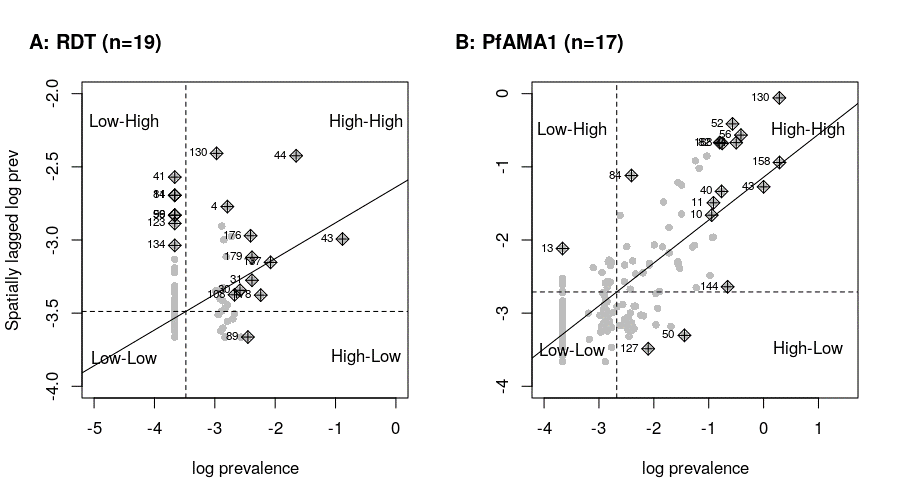


**Fig. S5**  Moran Scatterplots of clusters and hotspots of (A) malaria infection prevalence by RDT, and (B) PfAMA1 Ab seroprevalence.


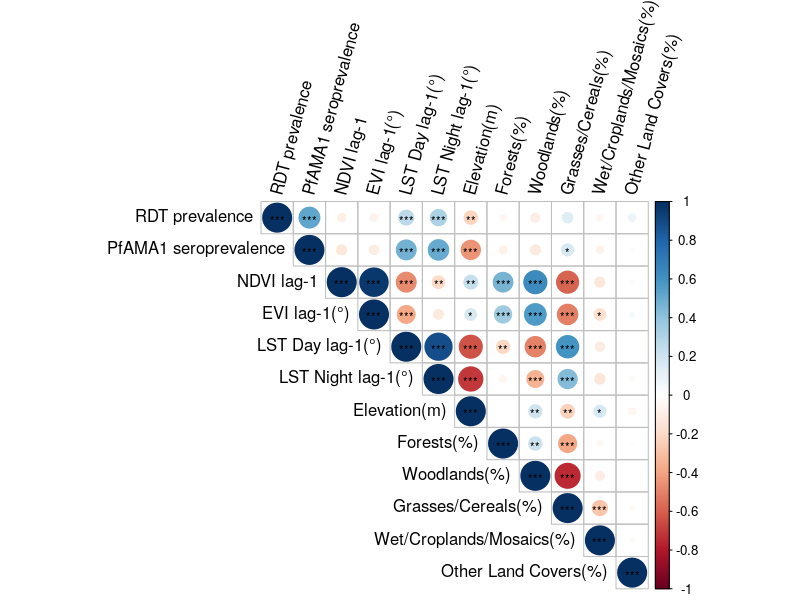


**Fig. S6** Pearson’s correlation scatterplot and peer’s significance of *P. falciparum* infection prevalence by RDT, PfAMA1 Ab seroprevalence and quantitative environmental and climatic covariates. Levels of significance are marked with (***) for p<0.001, (**) for p<0.01, and (*) for p<0.05. Lagged values of temperature and vegetation at 2 and 3 months were less correlated to malaria infection prevalence by RDT and PfAMA1 Ab seroprevalence than one-month lag at fokontany-level .


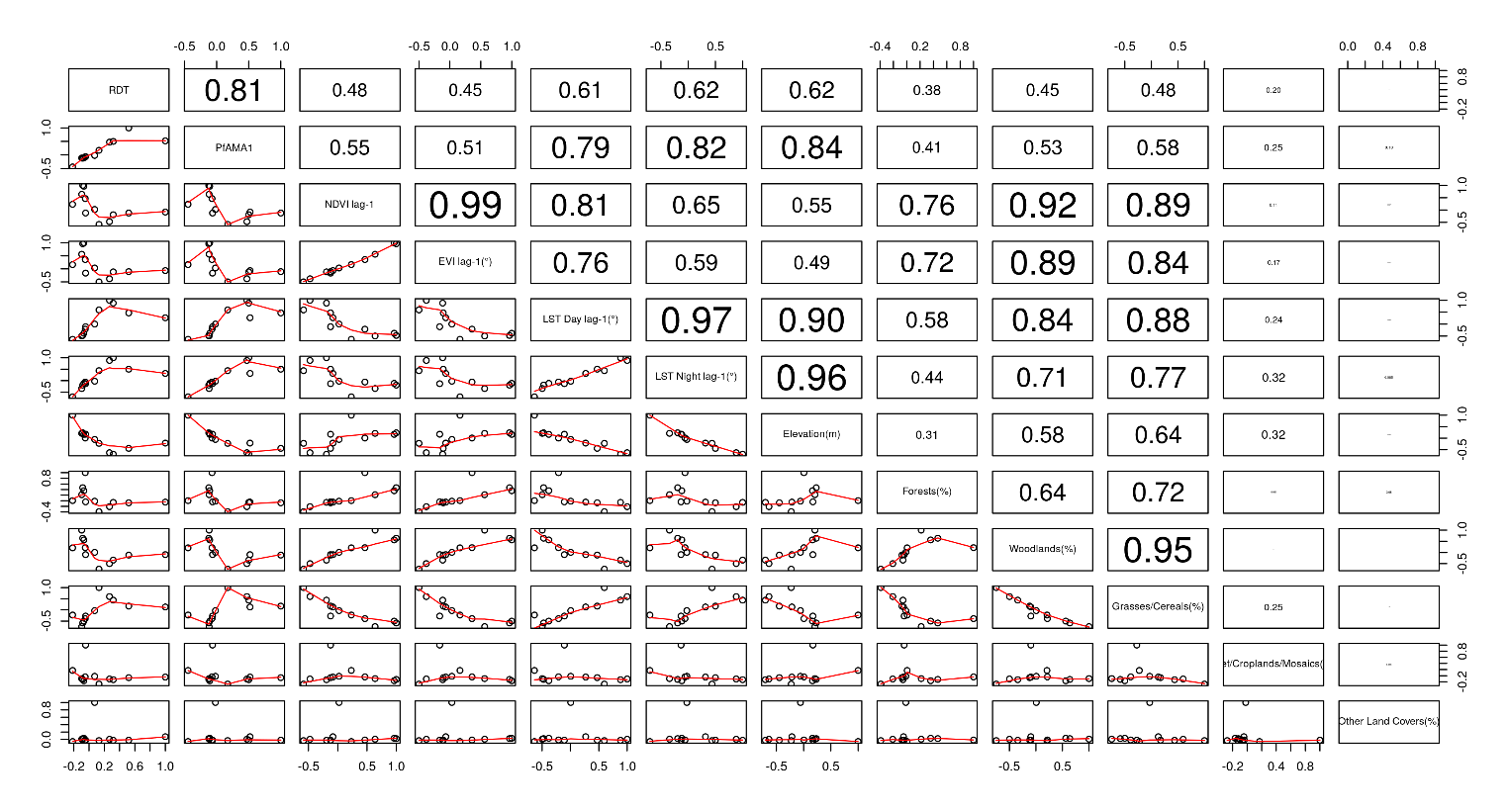


**Fig. S7**  Pearson’s correlation scatterplot and peers of *P. falciparum* infection prevalence by RDT, PfAMA1 Ab seroprevalence and quantitative environmental and climatic covariates associations. There was high correlation between multiple pairs of covariates such as {NDVI, EVI, forests, woodlands, or grasslands/cereals}, {LST day, LST night, elevation, woodlands or grasslands/cereals} and {grasslands/cereals, wet/croplands/mosaics}

**Table S2**  Univariable and multivariable *P. falciparum* exposure (PfAMA1 Ab response) model of individual-, household- and fokontany-level covariates, using mixed-effect logistic regression at district and commune level

| **Factors associated** | ***P. falciparum* exposure (PfAMA1 Ab response) model** | | | |
| --- | --- | --- | --- | --- |
|  | **Univariable** | | **Multivariable** | |
|  | **OR** | **95% CI** | **OR** | **95% CI** |
| **Individual level** |  |  |  |  |
| Age (years) |  |  |  |  |
| 2**–**8 (Ref.) | 1 |  | 1 |  |
| 9**–**10 | 1.5 | 1.1–2.0 | 1.8 | 1.2–2.4*** |
| 11**–**12 | 2.8 | 2.2–3.7 | 3.7 | 2.8–5.0*** |
| 13**–**14 | 4.0 | 2.9–5.4 | 5.7 | 4.0–8.0*** |
| Sex |  |  |  |  |
| Female (Ref.) | 1 |  |  |  |
| Male | 1.3 | 1.1–1.6 | 1.2 | 1.0–1.5 |
| Fever last 2 weeks | 1.7 | 1.3–2.3 | 1.7 | 1.2–2.4** |
| Trip last 2 months | 1.0 | 0.6–1.7 |  |  |
| Last night use of ITN | 2.0 | 1.7–2.5 |  |  |
|  |  |  |  |  |
| **Household level** |  |  |  |  |
| No. of ITN |  |  |  |  |
| [0,1] | 1 |  |  |  |
| (1,4] | 2.7 | 2.1–3.4 |  |  |
| (4,10] | 4.3 | 2.7–7.0 |  |  |
| Presence of RDT positive household member | 5.5 | 2.3–13.4 |  |  |
|  |  |  |  |  |
| **Fokontany level** |  |  |  |  |
| Health Facilities >5 km | 2.1 | 1.7–2.6 | 1.6 | 1.2–2.1** |
| School RDT prevalence^κ^ | 4.4 | 3.5–5.6 | 2.1 | 1.3–3.3** |
| NDVI at lag-1^η^ | 0.8 | 0.7–0.9 | 1.0 | 0.8–1.3 |
| LST Day at lag-1^¶^ | 4.5 | 3.7–5.4 |  |  |
| Grasslands or cereals^κ^ | 1.1 | 1.1–1.2 | 0.9 | 0.9–1.0 |
| Forests^κ^ | 0.8 | 0.7–1.0 |  |  |
| Woodlands^κ^ | 0.9 | 0.8–0.9 |  |  |
| Wet, croplands or mosaics^κ^ | 0.9 | 0.8–1.0 |  |  |
| Elevation^ε^ | 0.7 | 0.6–0.7 | 0.7 | 0.6–0.8*** |
| \| NDVI: Normalized Difference Vegetation Index at the previous month \| \| --- \| \| LST: daytime Land Surface Temperature and emissivity composites at the previous month \| \| ^η^ NDVI was scaled 1/10, as one unit increase means 0.1 increase \| \| \| ¶ LST Day was scaled in 5 °C unit, as one unit increase means 5 ° C increase \| \| \| κ Variables scaled in 10% unit, as one unit increase means 10% increase \| \| \| ε Elevation scaled in 100 m, as one unit increase means 100 m increase \| \|   OR: odds ratio; *** p-value <0.001; ** p-value < 0.01; * p-value < 0.05; Wald-test approximation was used for CIs (confidence interval) and p-values | | | | |
| Ref.: Reference; ITN: Insecticide-impregnated mosquito-nets | | | | |


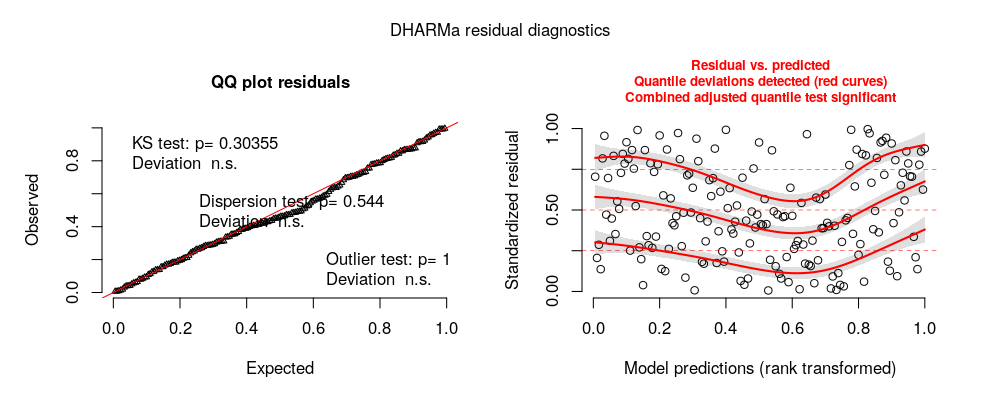


**Fig. S8** *P. falciparum* exposure (PfAMA1 Ab response) model residuals diagnostics of normality at fokontany-level. No significant deviance to normal distribution was observed PfAMA1 Ab response model residuals using Kolmogorov–Smirnov normality test (p = 0.3).


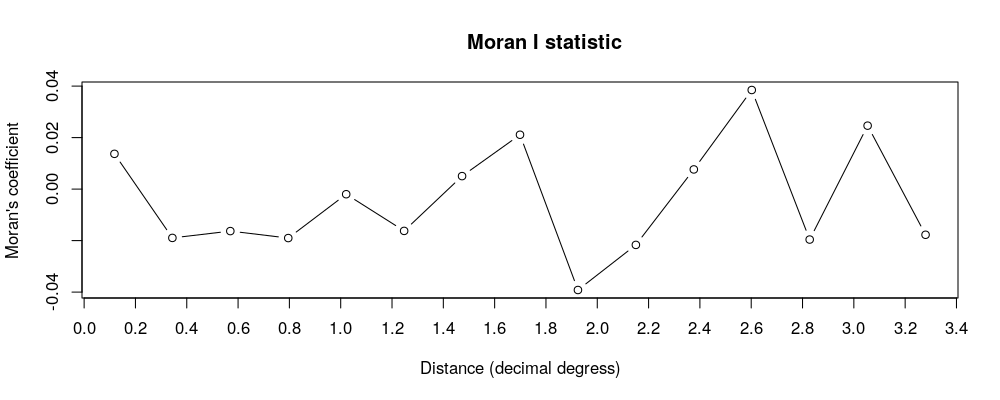


**Fig. S9** Spatial autocorrelation of residuals diagnostics using Moran’s I index of non-spatial *P. falciparum* exposure (PfAMA1 Ab response) model at fokontany-level.

**Table S5**  Univariable and multivariable *P. falciparum* infection model at individual-, household- and fokontany-level covariates using mixed-effect logistic regression at district and commune level

| **Factors associated** | ***P. falciparum* infection model** | | | |
| --- | --- | --- | --- | --- |
|  | **Univariable** | | **Multivariable** | |
|  | **OR** | **95% CI** | **OR** | **95% CI** |
| **Individual level** |  |  |  |  |
| Age (years) |  |  |  |  |
| 2**–**8 (Ref.) | 1 |  |  |  |
| 9**–**10 | 0.8 | 0.4–1.7 |  |  |
| 11**–**12 | 1.2 | 0.6–2.3 |  |  |
| 13**–**14 | 1.3 | 0.5–3.0 |  |  |
| Sex |  |  |  |  |
| Female (Ref.) | 1 |  |  |  |
| Male | 1.3 | 0.8–2.3 |  |  |
| Fever last 2 weeks | 8.9 | 5.1–15.6 | 9.9 | 5.2–18.9*** |
| Trip last 2 months | 0.6 | 0.1–4.0 |  |  |
| Last night use of ITN | 1.5 | 0.9–2.6 |  |  |
|  |  |  |  |  |
| **Household level** |  |  |  |  |
| No. of ITN |  |  |  |  |
| [0,1] | 1 |  |  |  |
| (1,4] | 2.21 | 1.2–4.1 |  |  |
| (4,10] | 1.2 | 0.2–9.3 |  |  |
|  |  |  |  |  |
| **Fokontany level** |  |  |  |  |
| Health Facilities >5 km | 1.8 | 1.1–3.1 |  |  |
| NDVI at lag-1 ^η^ | 0.7 | 0.5–0.9 |  |  |
| LST Day at lag-1^¶^ | 38.1 | 11.2–129.6 | 118.0 | 10.4–1.3e+03*** |
| Grasslands or cereals^κ^ | 1.3 | 1.1–1.6 |  |  |
| Forests^κ^ | 0.7 | 0.3–1.5 |  |  |
| Woodlands^κ^ | 0.7 | 0.6–0.9 | 1.1 | 0.8–1.5 |
| Wet, croplands or mosaics^κ^ | 0.8 | 0.5–1.3 |  |  |
| Elevation^ε^ | 0.7 | 0.6–0.8 |  |  |
| \| NDVI: Normalized Difference Vegetation Index at the previous month \| \| --- \| \| LST: daytime Land Surface Temperature and emissivity composites at the previous month \| \| ^η^ NDVI was scaled 1/10, as one unit increase means 0.1 increase \| \| \| ^¶^ LST Day was scaled in 5 °C unit, as one unit increase means 5 ° C increase \| \| \| ^κ^ Variables scaled in 10% unit, as one unit increase means 10% increase \| \| \| ^ε^ Elevation scaled in 100 m, as one unit increase means 100 m increase \| \|   OR: odds ratio; *** p-value <0.001; ** p-value < 0.01; * p-value < 0.05; Wald-test approximation was used for CIs (confidence interval) and p-values | | | | |
| Ref.: Reference; ITN: Insecticide-impregnated mosquito-nets | | | | |

**Table S6**  Sub-model for $\leq$5 years old children (seroprevalence = 2.6% [5 of 194]), only variables included in the all participants Ab response model were included: univariable *P. falciparum* exposure (PfAMA1 Ab response) model of individual- and fokontany-level covariates, using binomial logistic regression

| **Factors associated** | ***P. falciparum* exposure (PfAMA1 Ab response) model** | |
| --- | --- | --- |
|  | **Univariable** | |
|  | **OR** | **95% CI** |
| **Individual level** |  |  |
| Sex |  |  |
| Female (Ref.) | 1 |  |
| Male | 0.4 | 0.0–3.7 |
| Fever last 2 weeks | 1.9 | 0.2–17.8 |
|  |  |  |
| **Fokontany level** |  |  |
| Health Facilities >5 km | 2.0 | 0.3–12.4 |
| School RDT prevalence^κ^ | 0.8 | 0.0–52.1 |
| NDVI at lag-1^η^ | 2.1 | 0.9–5.0 |
| Grasslands or cereals^κ^ | 0.8 | 0.7–1.0 |
| Elevation^ε^ | 1.2 | 0.7–1.9 |
| \| NDVI: Normalized Difference Vegetation Index at the previous month \| \| --- \| \| ^η^ NDVI was scaled 1/10, as one unit increase means 0.1 increase \| \| \| ^κ^ Variables scaled in 10% unit, as one unit increase means 10% increase \| \| \| ^ε^ Elevation scaled in 100 m, as one unit increase means 100 m increase \| \|   OR: odds ratio; *** p-value <0.001; ** p-value < 0.01; * p-value < 0.05; Wald-test approximation was used for CIs (confidence interval) and p-values | | |
| Ref.: Reference; ITN: Insecticide-impregnated mosquito-nets | | |
